# Mental Fatigue in Parkinson's Disease: Systematic Review and Evaluation of Self-Reported Fatigue Scales

**DOI:** 10.1155/2024/9614163

**Published:** 2024-06-06

**Authors:** Junle Chen, Yanjun Zhou, Hengyi Rao, Jianghong Liu

**Affiliations:** ^1^Department of Neuroscience, School of Arts and Sciences, University of Pennsylvania, Philadelphia, PA 19104, USA; ^2^Department of Neurology, University of Pennsylvania, Philadelphia, PA 19104, USA; ^3^Department of Family and Community Health, School of Nursing, University of Pennsylvania, Philadelphia, PA 19104, USA

## Abstract

Fatigue is a common and debilitating symptom affecting a significant proportion of individuals with Parkinson's disease (PD), often overshadowing even motor symptoms in its impact on quality of life. The accurate definition and assessment of mental fatigue in PD is crucial for both clinical management and research, yet it remains a challenge due to the subjective nature of the symptom and the heterogeneity of assessment scales. This systematic review examined the existing measures of self-reported mental fatigue in PD by searching through PubMed, Embase, and Scopus databases using specific keywords from 2001 to 2024. Out of the 4182 articles found, 40 met the inclusion criteria, and 14 different scales were identified to measure self-reported fatigue in PD patients. However, most of these scales lack a consistent definition of fatigue, indicating a need for validated combinations of unidimensional and multidimensional scales to accurately assess mental fatigue in PD. The review found that it is best to use Fatigue Severity Inventory (FSI) and Multidimensional Fatigue Inventory (MdFI) to screen for severity of PD mental fatigue and Neuro-QoL Item Bank v1.0 (Neuro-QoL) to evaluate its impact on patients' lives. Furthermore, multidimensional scales Parkinson's Disease Questionnaire (PDQ) and Functional Assessment of Chronic Illness Therapy-Fatigue Scale (FACIT-F) are frequently coupled with Fatigue Severity Scale (FSS), Parkinson's Fatigue Scale (PFS), and/or Modified Fatigue Impact Scale (MFIS) due to their short length and holistic coverage of variables in patients' quality of life. Combining fatigue scales can be used for screening and scoring methods. The review also recommends validating fatigue scales translation and combining them with biomarkers to improve the accuracy and effectiveness of fatigue assessment in clinical practice. Future research should analyze correlations between fatigue scales, expand language types, and explore the link between fatigue scales and the pathophysiological basis of PD. Our findings underscore the need for a standardized approach to the measurement of fatigue in PD and set the stage for future research to consolidate assessment tools that can reliably guide treatment strategies and improve patient outcomes.

## 1. Introduction

Parkinson's Disease (PD) is the second most prevalent age-related neurodegenerative disorder and is primarily resulting from the death of dopaminergic neurons in the substantia nigra [1]. Recent data has shown that cases of PD increased by 155.51% during 1990 to 2019 [2]. PD is characterized by motor and nonmotor symptoms [3]. Motor symptoms, including gait disturbance, micrographia, precision grip impairment, and speech problems are known to be the central core of PD and are essential for clinical diagnosis, while nonmotor symptoms, including sleep disturbance, fatigue, abnormalities of sensation, and depression, are often neglected throughout the disease course [1, 4, 5]. The high prevalence and consequent disorder of PD raise the attention on nonmotor symptoms and highlight the importance of efficient diagnosis and targeted treatments.

As a nonmotor symptom of PD, mental fatigue, also known as Parkinson's apathy that conveys the subjective experience of feeling tired and difficulty to concentrate during an intellectual task, has not received as much attention as motor symptoms as a diagnosed factor for PD [6]. With severities comparable to motor symptoms, mental fatigue may also be present as a transient or persistent feature of PD [7]. Recent systematic reviews have provided evidence to demonstrate that fatigue affects PD patients more than age- and gender-matched controls [8]. For instance, approximately 50% of PD patients with either mild or severe motor fatigue due to various degrees of motor function hindrances have complained about mental fatigue [9]. Therefore, a good and appropriate validated measurement for mental fatigue is needed for diagnosis of PD. However, though current empirical studies have identified available measures on fatigue including Parkinson's Fatigue Scale (PFS), Fatigue Severity Scale (FSS), and Multidimensional Fatigue Inventory (McFI), little to no study has systematically reviewed and evaluated subjective measurements of fatigue in PD [10].

The overall goal of the review is to identify and evaluate the current fatigue measurements in the context of PD. This aim is three-folded: by examining empirical research on mental fatigue in PD, we hope to (i) identify prevalent subjective measurements for evaluating fatigue in patients with PD, (ii) compare and contrast current measurements from multiple aspects, and (iii) determine the specific utility and implication of these measurements. As fatigue is increasingly identified as a poorly recognized and inadequately treated symptom of PD, understanding the strengths, weaknesses, and correlations between current measurements of fatigue, namely, how different combinations of instruments can be used in varying scenarios, will aid future studies in improving diagnosis and treatments for PD [11].

## 2. Methods

### 2.1. Identification

**Figure 1:**
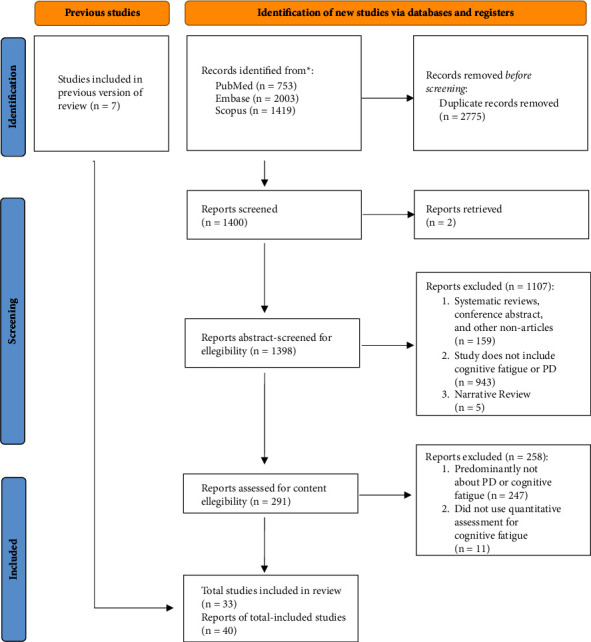
PRISMA (Preferred Reporting Items for Systematic Reviews and Meta-Analyses) flow diagram of the article selection process.

A systematic literature search was performed to retrieve published studies that included subjective measures for self-reported fatigue in patients with PD. The current review was conducted according to the PRISMA (Preferred Reporting Items for Systematic Reviews and Meta-Analyses) guideline, with specific steps shown in the PRISMA flow diagram in Figure 1 [12]. We searched literature in Scopus, PubMed, and Embase from 2001 to 2024 with keywords “fatigue” AND “Parkinson” AND (“scale” OR “questionnaire” OR “measure”).

### 2.2. Inclusion and Exclusion Criteria and Article Screening

Studies relevant to our research questions were expected to include measurements of fatigue in patients with Parkinson Disease. Specifically, the term “fatigue” in this study pertains to only mental and central fatigue, namely the subjective experience of exhaustion and weariness when initiating or performing a challenging task [6]. The term “measurements” comprises any systematic means of quantifying cognitive fatigue, mostly appearing in forms of scales and questionnaires. Our inclusion criteria for the studies that: (a) include and primarily focus on mental fatigue in patients with PD, (b) use systematic and quantitative means of quantifying cognitive fatigue, such as scales and questionnaires, to measure mental and central fatigue, and (c) articles published in or translated to English and have full-text availability. The year restriction was set to 2001–2024 in the literature search to focus on the most current and relevant scales that reflect modern practices and conceptions of mental fatigue in PD. By selecting studies that concentrate primarily on mental fatigue within PD patient demographic, the research aims to be honed to yield specific insights into this symptom. Requiring these studies to employ systematic and quantitative tools guarantees a level of consistency and objectivity across data sets. Such standardized methods both facilitate the comparison of findings and lend credibility to the review's conclusions.

Based on these definitions, we decided to screen all preliminary database results in two steps: abstract-screened eligibility and full-text reviewed eligibility. For the abstract screening, we exclude articles if they met any of the following criteria: (a) nonarticles (e.g., systematic reviews, meta-analyses, conference abstracts, unpublished thesis or dissertation), (b) does not include mental fatigue, and (c) narrative literature reviews or case study. This serves as a preliminary screening that ensures articles remain relevant and rigorous to our core research question, thus excluding articles with synthesized data that could skew the current review's findings, are mainly unrelated to the fatigue of interest, or have narrow focus. For the full-text content review, articles were excluded if they met any of the following criteria: (d) were predominantly not about PD or have less than one paragraph discussing mental fatigue, (e) did not use quantitative assessment for mental fatigue. All data were extracted via EndNote 20. A total of two reviewers were involved in the independent screening process.

### 2.3. Data Quality Assessment and Analysis

Studies were then assessed for research quality and reliability using the validity questions of the American Dietetic Association's Quality Criteria Checklist for primary research (Table 1). Studies are assessed on clear research questions, participant selection free of bias, comparable study groups, use of blinding, detailed intervention protocol, clearly defined outcomes with valid and reliable measurements, appropriate statistical analysis, results supporting the conclusions, and absence of funding bias. The overall quality rating is given as positive, neutral, or negative based on the number of “yes” responses to these criteria, with a focus on certain key aspects like participant selection, comparability of study groups, outcome clarity, and statistical analysis. Since the current study targets patients with PD, the assessment checkpoint “withdrawal or response rate” is not applicable and removed from the criteria.

The descriptive of scales used to evaluate fatigue in extracted studies were categorized based on the number of original studies that used each scale, the original language of the scale, the type of scale, the cutoff point for determining significant fatigue, the dimensions of fatigue assessed, the time required to administer the scale, the population for which the scale was developed, the method of assessment, and specific content or questions within each scale (Table 2). The descriptive aim is to provide an overview of the different scales available for measuring fatigue, how they are used, and their effectiveness across different studies and populations.

The reliability and validity of fatigue measures are assessed via psychometric evaluations (Table 3). Each scale was analyzed for internal consistency, with Cronbach's alpha values reported, and test-retest reliability to ensure measurement stability over time. The internal consistency is considered good if *α* ≥ 0.70, satisfactory if *α* ≥ 0.80, and desirable if *α* ≥ 0.90 [65]. Validity was appraised through correlation coefficients, affirming each scale's ability to accurately capture fatigue as a construct. Cronbach's alpha has been used to demonstrate the reliability of each scale. Implementation and adaptability of various fatigue scales were analyzed via languages that the scales have been translated, the associated neurobiological mechanisms, and the frequency of use in combination with other scales (Table 4). The combination of analysis on the reliability, validity, and applicability of the scales aim to demonstrate the scales' interdisciplinary relevance and adaptability alongside their rigor.

## 3. Results

From database searches based on selected keywords, PubMed, Embase, and Scopus yielded respectively 753, 2003, and 1419 results, a total of 4,175 articles. As shown in the PRISMA flow diagram in Figure 1, after identifying and removing duplicates and retrieving reports via EndNote 2.0, we derived 1400 relevant articles. From abstract screening, we exclude a total of 1075 articles that are either nonarticles (*n* = 159), irrelevant to mental fatigue (*n* = 943), or are narrative literature reviews or case study (*n* = 5). From the full-text content review (*n* = 291), we excluded a total of 177 articles that are mainly not relevant to PD or mental fatigue (*n* = 247) or did not use qualitative assessment for mental fatigue (*n* = 11). After including relevant studies from the previous version of the review (*n* = 7), a final sample of 40 articles assessing the subjective measurements of self-reported fatigue in PD was identified for the review.

As shown by the quality assessment of studies in Table 1, out of 40 articles assessed for quality, 27 were rated positively and 13 negatively, indicating that more than half of the articles are reliable for inclusion in the review We have found all studies to have clear research questions and reliable outcome with appropriate statistical analysis, with a vast majority of the studies having comparable study group and conclusion supported by results. However, alongside small sample sizes, the use of blinding and randomization was often not reported, indicating a potential methodological weakness and sources of sampling bias. The least favorable criterion was the potential for funding bias, with several studies being unclear or negative. The 40 reviewed articles targeted PD patients with a mean age of 60 to 70 years old from Europe (Norway (*n* = 1), Sweden (*n* = 1), and United Kingdom (*n* = 1), Netherlands (*n* = 1), France (*n* = 1), Spain (*n* = 2), Italy (*n* = 3), Slovakia (*n* = 2)), North America (United States of America (*n* = 7)), South America (Brazil (*n* = 3)), and Asia (Japan (*n* = 3), Philippines (*n* = 1), Vietnam (*n* = 1), South Korea (*n* = 1), China (*n* = 5), Turkey (*n* = 3), Iran (*n* = 1), and India (*n* = 3)).

Fourteen fatigue measurements were identified in the existing literature, with the specific descriptives listed in Table 2. FSS was the most commonly utilized, employed in 21 studies, and is a unidimensional scale focusing on fatigue intensity. It contrasts with the PFS used in 17 studies, which also measures physical and cognitive aspects of fatigue. Both scales require approximately 5 minutes for administration and are self-assessed. Other instruments like the MFIS and the MdFI were employed in fewer studies (8 and 5, respectively) and cover multiple dimensions of fatigue, including physical, cognitive, and psychosocial aspects. Most scales are in English, with difference in their cutoff points for determining significant fatigue that either uses a mean score or a percentile score. Scales vary in the dimensions of fatigue they assess, with some scales providing a more comprehensive evaluation than others. The administration time ranges from 5 to 20 minutes, indicating a consideration for the burden on respondents. The population for which each scale was developed also varies, including those with chronic illnesses such as Multiple Sclerosis, PD, and cancer. The detailed descriptions on all the scales' information can be found in the appendix.

In Table 3, the reliability and validity of all fatigue measurements were assessed. FSS, PFS, and PDQ were recognized as the top three frequently used fatigue measures. Specifically, FSS showed high internal consistency (*α* = 0.961), with discriminant validity shown through its ability to differentiate idiopathic PD (IPD) patients based on certain criteria. PFS demonstrated strong convergent validity with FSS and other scales, with internal consistency ratings (*α* = 0.96). PDQ exhibited good test-retest reliability and strong correlations between its items and factor loadings, suggesting strong construct validity. MFIS indicated strong correlations with various measures like PANAS-X and STAI-State, indicating its convergent validity. MdFI revealed strong internal consistency across its subscales and structural validity through PCA results. VAS-F presented strong test-retest reliability postexercise and significant correlations between its fatigue and energy subscales. Overall, scales show varying degrees of reliability and validity, suggesting different effectiveness in assessing fatigue in different contexts.

In Table 4, we have found that scales were coupled in two ways: unidimensional and bidimensional scales were used with multidimensional scales or unidimensional and bidimensional scales were coupled to other scales of the same dimension. Fatigue measurement scales have mostly been widely adopted across linguistic boundaries. Notably, FSS is the most translated and adapted scale, with versions available in multiple languages, indicating its global utility in assessing fatigue. Combinations of FSS, PDQ, and PFS are most frequently employed together in studies. For combinations of multidimensional and unidimensional scales, MFIS is employed most often with FSS, PDQ, or PFS.

## 4. Discussion

The goal of the study, namely, to identify and evaluate the current fatigue measurements in the context of PD, is three-folded: by examining empirical research on mental fatigue in PD, we hope to (i) identify prevalent subjective measurements for evaluating fatigue in patients with PD, (ii) compare and contrast current measurements from multiple aspects, and (iii) determine specific utility and implication of these measurements. Through a comprehensive systematic literature search performed across key databases such as Scopus, PubMed, and Embase, from 2001 to 2024, the current review captured the most recent and relevant studies related to mental fatigue and PD. A structured evaluation of the included studies' reliability and validity was conducted using the American Dietetic Association's Quality Criteria Checklist. Extensive analysis was carried out on the descriptive data extracted from the studies, particularly focusing on the scales used to evaluate fatigue, their reliability and validity, and the instruments' applicability across different contexts. By analyzing the methodology and the studies descriptives, the current study identifies and evaluates a total of 14 fatigue scales for their ability to measure mental fatigue in PD patients. Upon reviewing all scales, there is a universal lack of explicit definition for fatigue within most of the scales. Basing the scale quality assessment on that premise, the scale evaluation of convergent validity and reliability suggest that the usage of solely one scale is insufficient for diagnosing mental fatigue in PD, thus needing validated combinations of unidimensional and multidimensional scales. Specifically, due to the characteristics of scale questions and scoring methods, fatigue measurements can be used combinatorially for screening and in-depth assessment. Our findings also demonstrate continuing validity of scales upon translation and potential correlation between mental fatigue and neural mechanisms.

### 4.1. Lack of Explicit Definition for Fatigue within Scales

Upon collecting data from and analyzing fatigue-measuring scales of PD, there is a prevalence of either lack of or inconsistent definition for fatigue. As shown by many previous scholarships, fatigue is multidimensional due to its perception based on subjective observations and various subdomains: physical, mental, peripheral, and central fatigues. As defined by Krupp in his book about fatigue, physical fatigue can be defined as a subjective phenomenon pertaining to perceptions of exhaustion and deprivation of energy to perform physical tasks despite physical capability and motivation to do such. Comparatively, mental fatigue is defined as the sense of exhaustion and lack of energy experienced after performance of subjective demanding cognitive tasks that require elevated levels of focus [67]. Chaudhuri defined peripheral fatigue, the anatomical explanation of physical fatigue, as the decrement in movement frequency or performance caused by muscle fatigue, while central fatigue, the basis of mental fatigue, as the perception of feeling abnormally exhausted given the normal levels of concentration [68]. Given these distinctly different definitions, the inconsistent and lack of definition for fatigue among measurements would skew the scales' psychometric performances in absence of a categoric biological and clinical understanding of mental fatigue. For instance, fatigue symptoms pertaining to characteristics of both physical and mental fatigues would lead to confounding results and unclear trends.

Among the 14 scales that are extracted, reviewed, and evaluated, only FAI and FIS included a clear definition of fatigue and differentiated dimensions of fatigue in their questionnaires. Regardless of the objective anatomical basis of fatigues, perception differs across subjects due to diverse subjective definitions of cognitive exhaustion and interpretation of degrees of mental tiredness. Compounding such subjectivity with the varying weights different scales attach to separate subscales and individual items, absence of or inconsistent definition of fatigue would lower the validity of these instruments. Differing scale types may also increase the variation of fatigue measurements. Given the subjectivity of mental fatigue, categorical scales like NHP's dichotomous scale might convey different severity of fatigue compared to degree-varying scales like MFIS's Likert scale due to the subjective boundary for fatigue and scales' different cutoffs to determine fatigue. Thus, with the lack of biological markers to determine mental fatigue objectively, explicit definition of fatigue and related dimensions would lead to more valid results.

### 4.2. Evaluation of Scales for Mental Fatigue Measurements

Among the 14 fatigue scales identified from our final articles, the three most popular fatigue scales are FSS, PFS, and PDQ. However, upon further analyses and assessments on their psychometric properties and descriptive, we propose a different strategy in choosing the best scales specifically for mental fatigue measurements.

Within existing literature about PD fatigue measurement, most scales lack a consistent definition of fatigue, thus neglecting the minute differences between physical and mental fatigue. When current scholars screen PD fatigue severity and try to determine the effects that fatigue have on various aspects of patients' quality of life, the extracted information from these measurements would be about either general fatigue or solely physical fatigue (in the case of PFS). In addition, many scales, like MFIS and BFI, are not or insufficiently validated for PD. Thus, to evaluate the abilities of the 14 scales on assessing mental fatigue, a rough screening for discrimination between various subdomains of fatigue within scale and validation for PD, as demonstrated in Table 3, is needed. Differentiation for mental fatigue is determined by the explicit separation of items for mental and other fatigues within examined scales. Validation for PD is determined by the abundance of data on the scales' reliability and validity in PD.

Based on these definitions, only scales with “Yes” in both categories or “Vague” and “Yes” are eligible for evaluation, which effectively eliminates FSS, PFS, MFIS, VAS-F, McFI, BFI, FSI, and NHP. Among scales that are left, PDQ, MdFI, Neuro-QoL, and FACIT-F are multidimensional scales, while FIS is unidimensional. Consequently, if the research focus is to screen for the severity of PD mental fatigue, FSI and MdFI would rate highest due to reliability, and discriminant validity. However, if the focus is to determine the impact of varying degrees of mental fatigue on patients' life, then Neuro-QoL would be most optimal due to its holistic dimensions, strong internal consistency, and great convergent validity. After being selected based on intrascale correlations, the rest of the scales can be used to cross-examine results and explore varying degrees of mental fatigue's correlations with different dimensions of quality of life.

Despite their elimination from evaluation based on the current criteria, some scales can be used as preliminary screening tools to assess the severity of general fatigue within PD patients. For instance, FSS, NHP, and PFS can both be useful screening tools due to their brevity and ease to administer and understand. Once validated, MFIS, BFI, and VAS-F would also be useful screening tools given their excellent reliability and convergent validity with scales measuring many other aspects of PD-related variables. Scales like McFI and FSI should be removed from the list of fatigue measurements due to their lack of reliability and validity, scarcity of usage, and statistical insignificance.

### 4.3. Convergent Validity and Reliability in Fatigue Measures

Across almost all existing literature we examined, fatigue scales are used combinatorially to assess the multitude of PD symptoms, especially the impact of mental fatigue, psychological health and quality of life. This suggests that the application of only one scale is not sufficient for diagnosing mental fatigue in PD. Based on the occurrence of said combinations and convergent validities, there are strong correlations between the 4 most commonly used pairs: (1) FSS and PDQ; (2) FSS and PFS; (3) PFS and PDQ; and (4) PFS, FSS, and MFIS, demonstrating future studies could improve reliability and validity of PD fatigue diagnosis by using these pairs in addition to the specific utility of each scale. In the specific context of measuring mental fatigue, many new combinations are formed due to their strong correlations and convergent validity: (1) FSS, FACIT-F, and NHP; (2) PFS, PDQ, and FSS; (3) MdFI and FIS; (4) FAI and VAS-F; and (5) Neuro-QoL and all other unidimensional scales measuring fatigue severity. Though aforementioned options are the most prevalent combinations, scales can be used simultaneously due to the individual aspects of PD symptoms that each test specializes in measuring. Considering their different characteristics, scales can be coupled according to two research foci: varying degrees of physical and mental fatigue and their respective impacts on the various perspectives of PD patients' lives.

Among 14 fatigue measurements, 9 target fatigue specifically. The three most frequently used fatigue measures are FSS, PFS, and MFIS. They are preferable because of their short questionnaire length, low redundancy, and high accuracy. Other measures specializing in fatigue are MdFI, McFI, BFI, FAI, FIS, and FSI. Specific for mental fatigue, FIS and MdFI would be the two most appropriate scales measuring severity of mental fatigue due to reliability and validity. Among these scales, FAI, FIS, and FSI are all derived from FSS. Since FSS only contains nine questions, expanding items in the questionnaire could provide a more comprehensive analysis. As a result, FIS and FSI expand the questionnaire and are unidimensional as FSS. In contrast, FAI is multidimensional, with dimensions other than fatigue intensity. However, FSI, FIS, and FAI consume more time which PD patients may not prefer. Upon analyzing both convergent validity and reliability, the nine measures are all coupled with one to two scales, suggesting that individual scales would be insufficient in confirming trends regarding PD fatigue, and using multiple scales could provide cross-validation of results. However, coupling of more than two scales is rare due to the potential redundancy between scales.

To compound with unidimensional scales specifically aiming to test severity of physical and mental fatigue, multidimensional tests regarding various aspects of patients' lives, namely depression, anxiety, emotional and social wellbeing, pain, and quality of life, can be used concurrently with the unidimensional scales to probe the holistic impacts of fatigue intensity on patients. All of the 5 fatigue measurements that test the multidimensional effects of PD fatigues have exceptional reliability. However, only PDQ and/or FACIT-F, are frequently coupled with FSS, PFS, and/or MFIS due to their short length and holistic coverage of variables in patients' quality of life. Specifically, scale's brevity and comprehensiveness would facilitate not only the administrators' quantification of data, but also PD patients' response to the questions, which would reduce the likelihood of data skewness caused by incomprehension and mental fatigue. Despite covering almost all domains of patients' quality of life, Neuro-QoL's long questionnaire length and administer time could lead to confounding trends during data analysis for impacts of fatigue and burden on subjects' cognition, both of which would tamper the accuracy of the results. However, this characteristic may be beneficial if the emphasis of quantification is placed on the holistic impacts of PD symptoms rather than solely fatigue. In context of assessing mental fatigue in such a research focus, Neuro-QoL should be coupled with FSI and/or MdFI due to strong correlation and internal consistency. Another strong combination would be FIS and MdFI due to their tested convergent validity.

### 4.4. Application of Fatigue Measurements

The findings show that almost every fatigue scale discussed in our review recognizes English as the original language except for MdFI. This indicates that prevalent fatigue measurements are targeting mainly English speakers. However, many of these instruments can be used to determine the severity of patients' mental fatigue in relation to the development of PD symptoms [69]. PFS has the most language modifications, including Japanese, Spanish, Turkish and Chinese. FSS has been tested valid in the Portuguese and Turkish versions. MFIS has shown modifications in Brazilian and Portuguese versions. McFI has been validated in Dutch and FIS in Turkish versions. Considering the prevalence of fatigue in PD, our findings shed light on the potential language modifications of fatigue scales. The results indicate that FSS, PFS, MFIS, McFI, and FIS have been proven to be valid and reliable after translating into nonoriginal languages, which could be developed as standard scales internationally and facilitate other non-English speaking countries to enhance PD fatigue diagnosis.

Though the possible pathophysiological basis of mental fatigue in PD has not been addressed in our current systematic review, based on our robust findings, some speculation is warranted. In particular, our results demonstrate that FSS targets motor symptoms, cortical function, and autonomic dysfunction. In contrast, PFS and MFIS are specifically utilized in measuring fatigue in studies relating to the subthalamic nucleus in PD patients. Furthermore, one study suggested that BFI can lead to the prediction of hallucinations, which is a risk and imperceptible factor for PD [24]. By identifying the correlation between mental fatigue and hallucinations, mental fatigue can be used to indicate the sign of hallucinations and thereby determine PD. Though the study did not directly measure fatigue, the indirect use of fatigue to measure other undetectable and severe factors in PD would be a potential and comprehensive diagnostic tool for future studies.

### 4.5. Limitations

Although we used a comprehensive literature search and thoroughly explored our data, the current review still has several potential limitations. First, we excluded studies that were not written in English or did not have English translations, which may have caused language bias. Studies published in other languages might provide insights on performance of scales in diagnosing PD fatigue. Second, the review did not identify all cutoff points due to the unavailability of relevant data, so confounding variables in the fatigue scale itself could not be avoided. Another limitation is that since our final sample size of relevant articles are small; there could be other studies on the correlation between certain fatigue scales that we did not cover. Finally, we did not compare the quantitative reliability of scales, which could have affected the respective results.

## 5. Conclusion

In conclusion, the assessment of mental fatigue in PD requires the use of multiple fatigue scales that are validated and specific to the subdomain of fatigue being measured. The lack of a consistent definition of fatigue in many scales highlights the need for a screening for discrimination between various subdomains of fatigue within the scale and validation for PD. FSI and MdFI rate highest for screening for the severity of PD mental fatigue, while Neuro-QoL is optimal for determining the impact of varying degrees of mental fatigue on patients' lives. Additionally, preliminary screening tools such as FSS, NHP, and PFS can be used to assess the severity of general fatigue within PD patients. Coupling fatigue scales with unidimensional and multidimensional scales that measure various aspects of patients' lives provides a more comprehensive analysis of the holistic impacts of fatigue intensity on PD patients. Among the multidimensional scales, PDQ and FACIT-F are frequently coupled with FSS, PFS, and/or MFIS due to their short length and holistic coverage of variables in patients' quality of life. However, Neuro-QoL's long questionnaire length and administer time could lead to confounding trends during data analysis for impacts of fatigue and burden on subjects' cognition. In context of assessing mental fatigue, Neuro-QoL should be coupled with FSI and/or MdFI due to strong correlation and internal consistency, and FIS and MdFI due to their tested convergent validity. Future research should also focus on validating fatigue scales before translating them into non-English languages and combining fatigue scales with biomarkers to enhance the accuracy and efficacy of fatigue assessment in clinical practice.

### 5.1. Implications

#### 5.1.1. Implication for Future Research

The current review presents significant implications for future research on fatigue assessment in PD. Firstly, investigating the correlation between various fatigue scales could increase diagnostic accuracy. Specifically, studies should explore the sensitivity and differences between physical and mental fatigue measures across scales. To facilitate clinical application, future research should investigate the validity of each fatigue in PD, its convergence with other PD-related factors, and its specificity and sensitivity for individual patients. Given the inherent subjectivity of psychometric measurements, biomarker discovery may contribute to transforming current scales into objective measurements of multidimensional fatigue.

While existing fatigue scales have demonstrated validity and reliability in only 10 languages, further research is needed to examine the applicability of fatigue scales in other languages. Moreover, although each fatigue scale targets different dimensions of neurobiology mechanisms in PD, the relationship between these mechanisms and fatigue requires further empirical support. Additionally, to mitigate language bias, future studies could incorporate non-English language articles. Finally, more studies are warranted to determine clear cutoff points for each fatigue scale.

Taken together, these implications highlight promising avenues for future research to enhance fatigue assessment in PD. Addressing these issues may help optimize the diagnosis and management of fatigue in PD and ultimately improve patient outcomes.

#### 5.1.2. Implications for Future Practice

The analysis of fatigue scales and their psychometric properties has identified 10 reliable and appropriate tools for evaluating fatigue in PD patients, with 4 eliminated from the total 14 scales due to lack of reliability and validity, scarcity of usage, and statistical insignificance. FSS is particularly useful as a clinical screening tool to assess the severity of general fatigue among a large patient population. However, due to its ambiguous definition of fatigue, it is not well-suited for evaluating mental fatigue. PFS is a good screening tool for unidimensional physical fatigue but is not appropriate for evaluating multidimensional aspects of fatigue or clinical applications. MFIS is a valuable clinical tool for assessing chronic fatigue and its impact on comorbidities in PD, but not for PD symptoms. PDQ is a multidimensional tool that can evaluate the impact of fatigue on different aspects of a patient's life. MdFI is a useful clinical tool for screening and determining fatigue severity in a large PD patient population. VAS-F has a user-friendly design, but more research is needed to investigate its test-retest reliability in PD. FIS provides a brief yet comprehensive assessment of multicomponent fatigue and its impact. NHP is a screening tool that measures fatigue severity and its impact on various aspects of patients' quality of life. FAI is a holistic assessment of fatigue, particularly its impact on different aspects of daily life, and future research is needed to evaluate its validity in PD. FSI, Neuro-QoL, McFI, and BFI may not be reliable screening tools for assessing fatigue in PD patients due to limited data and psychometric properties.

These findings have significant implications for future practice. Clinicians should carefully consider the appropriate tools to use in evaluating fatigue, depending on their specific research or clinical goals. Further research is needed to validate and refine the existing fatigue scales, investigate their cross-cultural applicability, and explore biomarkers for objective measurement of fatigue. Such efforts may help enhance diagnosis, treatment, and management of fatigue in PD patients, ultimately improving their quality of life.

## Figures and Tables

**Table 1:** Quality assessment of selected articles^1^.

Study	(1) Clear research questions	(2) Participant selection free of bias	(3) Comparable study group	(4) Use of blinding	(5) Intervention protocol and/or data collection procedure described in detail	(6) Outcome clearly defined and the measurements valid and reliable	(7) Appropriate statistical analysis	(8) Conclusion supported by results	(9) Unlikely funding bias	Overall quality rating^2^
Friedman et al. [8]	Y	Y	Y	N	N	Y	Y	Unclear	Unclear	−
Alves et al. [13]	Y	Y	Y	N	Y	Y	Y	Y	Unclear	+
Havlikova et al. [7]	Y	N	Y	N	Y	Y	Y	Y	N	+
Goulart et al. [14]	Y	N	Y	N	N	Y	Y	Unclear	Unclear	−
Rabo et al. [15]	Y	N	Y	N	Y	Y	Y	Y	Unclear	+
Chou et al. [16]	Y	N	Y	N	Y	Y	Y	Y	N	+
Elbers et al. [17]	Y	Y	Y	Y	Y	Y	Y	Y	N	+
Valderramas et al. [18]	Y	Y	Y	N	Y	Y	Y	Y	N	+
Sáez-Francas et al. [19]	Y	N	N	N	N	Y	Y	Y	Unclear	−
Stocchi [20]	Y	Y	Y	Y	Y	Y	Y	Y	N	+
Tanaka et al. [21]	Y	N	Y	N	N	Y	Y	Y	N	−
Cochrane et al. [22]	Y	N	Y	N	Y	Y	Y	Y	Unclear	+
Skorvanek et al. [23]	Y	N	Y	Y	Y	Y	Y	Y	N	+
Ikeda et al. [24]	Y	N	Y	N	Y	Y	Y	Y	Y	+
Paul et al. [25]	Y	N	Y	N	Y	Y	Y	Y	Y	+
Tessitore et al. [26]	Y	Y	Y	Y	Y	Y	Y	Y	Unclear	+
Hidding et al. [27]	Y	Y	N	Y	Y	Y	Y	Y	N	−
Chong et al. [28]	Y	N	Y	N	Y	Y	Y	Y	Y	+
Olivola et al. [29]	Y	N	Y	Y	Y	Y	Y	Y	Unclear	+
Öztürk et al. [30]	Y	N	N	N	N	Y	Y	Y	Y	−
Yang et al. [31]	Y	N	Y	N	N	Y	Y	Y	Unclear	−
Atan et al. [32]	Y	Y	Y	Y	Y	Y	Y	Y	Unclear	+
Çilga et al. [33]	Y	N	Y	N	Y	Y	Y	Y	Unclear	+
Martinez-Martin et al. [34]	Y	N	Y	N	N	Y	Y	Y	N	−
Nguyen et al. [35]	Y	N	Y	N	Y	Y	Y	Y	Y	+
Niimi et al. [36]	Y	N	Y	N	Y	Y	Y	Y	N	+
Abasi et al. [37]	Y	N	Y	Y	Y	Y	Y	Y	N	+
Ahn et al. [38]	Y	N	Y	N	Y	Y	Y	Y	Unclear	+
Lopes et al. [39]	Y	N	N	N	Y	Y	Y	Y	Unclear	−
Singh et al. [40]	Y	N	Y	N	Y	Y	Y	Y	Unclear	+
Yu et al. [41]	Y	N	N	N	N	Y	Y	Y	N	−
Cao et al. [42]	Y	N	Y	Y	N	Y	Y	Y	N	−
Hill et al. [43]	Y	N	Y	N	Y	Y	Y	Y	N	+
Lee et al. [44]	Y	N	Y	N	Y	Y	Y	Y	N	+
Lin et al. [45]	Y	N	N	N	Y	Y	Y	Y	N	−
Alizadeh et al. [46]	Y	Y	Y	N	Y	Y	Y	Y	N	+
Paul et al. [47]	Y	N	Y	N	Y	Y	Y	Y	Y	+
Béreau et al. [48]	Y	N	N	N	Y	Y	Y	Y	N	−
Lawrie et al. [49]	Y	N	Y	N	Y	Y	Y	Y	N	+
Siciliano et al. [50]	Y	N	Y	Y	Y	Y	Y	Y	N	+

^1^Y, yes; N, no; unclear, not clearly reported. Positive (+): if most (six or more) of the answers to the above validity questions are “yes” (including 2, 3, 6, and 7), the report should be designated with a plus symbol (+), neutral (Ø): if answers to validity criteria question 2, 3, 6, and 7 are “yes” but several other criteria indicate study weakness, the report should be designated with a neutral symbol. Negative (−): if most (six or more) of the answers to the above validity questions are “no,” the report should be designated with a minus symbol. Since question 4 “withdraws or response rate described” is not applicable in this review, the study did not rate question 4. ^2^Among 40 articles, 13 articles are found to be negative, and 27 articles are found to be positive. More than half the articles are reliable for this review.

**Table 2:** Descriptive of fatigue scales^1^.

Instrument (abbreviation)	Number of studies	Original language	Scale type and number of items	Cutoff point	Dimension	Time to administer	Development population	Assessment method	Contents
Fatigue Severity Scale (FSS)	21	English	7-Point Likert scale: 9 items	Mean > 5 Mean ≥ 4 Mean > 4	Unidimensional Fatigue Intensity (physical and cognitive)	5 min	Multiple Sclerosis	Self-assessment	Has physical and mental fatigue questions:
									My motivation is lower when I am fatigued
									Exercise brings on my fatigue
									I am easily fatigued
									Fatigue interferes with my physical functioning

Parkinson's Fatigue Scale (PFS)	17	English	5-Point Likert scale: 16 items	Quantitative: Mean ≥ 3 Qualitative: Mean ≥ 8	Unidimensional Fatigue Intensity (physical and cognitive)	5 min	Parkinson's disease	Self-assessment	Has physical and mental fatigue questions:
									I have to rest during the day
									I feel completely exhausted
									I have a feeling of “heaviness”
									I lack energy for much of the time
									Fatigue makes it difficult for me to cope with everyday activities

The Parkinson's Disease Questionnaire (PDQ)	11	English	5-Point Likert scale: (i) Long form: 39 items (ii) Short form: 8 items	Percentile score	Multidimensional Fatigue Intensity (mobility, cognition) Quality of Life (activities of daily life, social support, communication, bodily discomfort) Depression (emotional well-being, stigma)	10–20 min	Parkinson's Disease	Self-assessment	Has physical and mental fatigue questions:
									Has difficulty doing the leisure activities which you would like to do?
									Hard problems walking half a mile?
									Felt anxious?
									Felt ignored by people?

Modified Fatigue Impact Scale (MFIS)	8	English	5-Point Likert scale: (i) Long form: 21 (ii) Short form: 5	38	Multidimensional Fatigue Intensity (physical and cognitive) Psychosocial functioning	2–10 min	Multiple Sclerosis	Self-assessment	Has physical and mental fatigue questions:
									I have been clumsy and uncoordinated
									I have to pace myself in my physical activities
									I have been less motivated to do anything that requires thinking
									My thinking has slowed down

Multidimensional Fatigue Inventory (MdFI)	5	Norwegian	5-point Likert scale: 20 items	N/A	Multidimensional	5–10 min	Chronic Fatigue Syndrome Cancer	Self-assessment	Has physical and mental fatigue questions:
					General fatigue (GF)				I feel fit
					Physical fatigue (PF)				I feel very active
					Mental fatigue (MF)				I feel tired
					Reduced motivation (RM)				I think I do a lot in a day
					Reduced activity				Physically I feel I am in an excellent condition

Visual Analog Scale for Fatigue (VAS-F)	2	English	10-point visual analogic scale: 18 items	Percentile	Bidimensional Fatigue Intensity (physical and cognitive) Energy	5–10 min	None specified	Self-assessment	Has physical and mental fatigue questions:
									Not at all tired-extremely tired
									Not at all energetic-extremely energetic
									Not at all exhausted-extremely exhausted
									I have absolutely no desire to close my eyes-I have a tremendous desire to close my eyes

Multicomponent Fatigue Index (McFI)	1	English	5-point Likert scale: (i) Physical fatigue: 7 items (ii) Cognitive fatigue: 8 items	N/A	Unidimensional Fatigue Intensity (physical and cognitive)	5–10 min	None specified	Self-assessment	Has physical and mental fatigue questions:
									Do you currently have problems concentrating?
									Are you feeling less motivated than usual?
									Are you having problems thinking clearly?
									Are you having problems with tiredness?
									Do you feel like you need to take a rest right now?
									Do you currently feel sleepy or drowsy?

Brief Fatigue Inventory (BFI)	1	English	11 (0–10)-point Likert scale: 9 items	36	Unidimensional Fatigue Intensity (physical and cognitive)	5 min	Cancer	Self-assessment	Has physical and mental fatigue questions:
									General activity
									Walking ability
									Mood
									Enjoyment of life

Fatigue Symptom Inventory (FSI)	1	English	11 (0–10)-point Likert scale: 14 items	42	Unidimensional Fatigue Intensity (physical and cognitive)	5–10 min	Cancer	Self-assessment	Has physical and mental fatigue questions:
									Rate level of fatigue on the day you felt most fatigued during the past week
									Rate how much, in the past week, fatigue interfered with your general level of activity
									Rate how much, in the past week, fatigue interfered with your ability to concentrate

Neuro-QoL Item Bank v1.0 (Neuro-QoL)	1	English	5-Point Likert Scale: (i) Adult Bank: 10–45 items (ii) Adult short form: 5–9 items (iii) Pediatric: 1–20 items (iv) Pediatric short form: 8–10 items	N/A	Multidimensional	2-3 min/domain	Stroke Multiple Sclerosis Parkinson's Disease Epilepsy Amyotrophic lateral sclerosis	Self-assessment Interview	Has physical and mental fatigue questions: I felt exhausted I was too tired to do my household chores I felt tired I had to limit my social activity because I was tired
					Fatigue intensity				
					Activities of daily living				
					Attention and working memory				
					Behavior				
					Cognition				
					Communication				
					Depression				
					Functional mobility				
					Mental health				
					Occupational performance				
					Life participation				
					Pain				
					Negative and positive effects				
					Quality of life				
					Sleep				
					Relationships				
					Stress				
					Social support				

Functional Assessment of Chronic Illness Therapy-Fatigue Scale (FACIT-F)	1	English	5-point Likert scale: 13 items	N/A	Multidimensional Fatigue Intensity (physical and cognitive) Well-being (emotional and social)	10–15 min	HIV/AIDS Multiple Sclerosis Parkinson's Disease Rheumatoid Arthritis	Self-assessment	Has physical and mental fatigue questions:
									I have a lack of energy
									I have nausea
									I feel ill
									I feel sad
									I feel nervous
									I worry about dying

Fatigue Impact Scale (FIS)	1	English/French	5-Point Likert scale: 40 items	N/A	Unidimensional Fatigue Intensity (physical and cognitive)	10–15 min	None specified	Self-assessment	Has physical and mental fatigue questions:
									Because of fatigue, I feel less alert
									Because of fatigue, I feel slowed down in my thinking
									Because of fatigue, I have to limit my physical activities

Nottingham Health Profile (NHP)	1	English	Yes/no dichotomous scale: 38 items	Weighted percentage	Multidimensional sleep	10 min	None specified	Self-assessment	Has physical and mental fatigue questions
					Physical mobility				I feel lonely
					Fatigue pain				I'm feeling on edge
					Emotional reactions				The days seem to drag
					Social isolation				I feel I am a burden to people

Fatigue Assessment Inventory (FAI)	1	English	7-point Likert scale: 29 items	N/A	Multidimensional Fatigue Intensity (physical and cognitive) Sleep Severity Situation-specificity Psychological consequences	5–10 min	None specified	Self-assessment	Has physical and mental fatigue questions:
									Exercise brings on my fatigue
									Heat brings on my fatigue
									Work brings on fatigue
									My motivation is lower when I am fatigued
									Stress brings on my fatigue
									Depression brings on my fatigue

^1^The characteristics of each scale are presented, including number of studies, original language, scale type, cutoff point, dimension, time to administer, development population, assessment method, and contents.

**Table 3:** Reliability and validity of fatigue measures^1^.

Scale	Article (first author)	Sample	Reliability	Validity	Mental fatigue subscale
Fatigue Severity Scale (FSS)	Fereshtehnejad [51] Hagell and Nilsson [52]	Idiopathic Parkinson's disease (IPD) patients that are (1) age 35 years +; (2) those with moderate to severe dementia MMSE <24 (*n* = 90) Patients who are (1) age 54–74; (2) Hoehn and Yahr stage I–III (*n* = 118)	Internal consistency (Iran): FSS total: *α* = 0.961, ICC (95% CI) = 0.948–0.972 (*p* < 0.001) Age, sex, educational level, and PD severity: *α* > 0.9 (*p* < 0.001) Internal consistency (Sweden): FSS total: *α* = 0.94	Spearman's rho >0.8 for all items (*p* < 0.001)	No
				Total score strongly correlated with fatigue severity (*r* = 0.548, *p* < 0.001), Hoehn and Yahr stages (*r* = 0.478, *p* < 0.001) and Schwab and England ADL scale (*r* = −0.487, *p* < 0.001)	
				Discriminant validity: FSS's ability to discriminate IPD patients with H and Y stage >2 (AUC = 0.81, 95% CI = 0.72–0.90, *p* < 0.001) and cutoff value = 4.5 is best diagnostic value (92.6% sensitivity and 61.9% specificity)	
				Floor and ceiling effects were minimal	
				Construct validity	
				Strongly correlated with FACIT-F (*r* = −0.77, *p* < 0.0001) and NHP-EN (0.62)	

Parkinson's Fatigue Scale (PFS-16)	Dagklis et al. [53]	Patients diagnosed according to the UK PD society brain bank diagnostic criteria for idiopathic PD (*n* = 99)	Internal consistency:	Spearman's rho (−0.71 to 0.77) are significant for all PD-related variables (*p* < 0.01) except for age and MMSE	No
			Original scoring: *α* = 0.96, ICC (95% CI) = 0.93 (0.81–0.97)	Convergent validity:	
			Binary scoring: *α* = 0.92, ICC (95% CI) = 0.93 (0.82 to 0.97)	PFS-16 is significantly correlated with FSS (*r* = 0.77, *p* < 0.001), SF-36-VT (*r* = − 0.70, *p* < 0.001), PDQ-8 (*r* = 0.65, *p* < 0.001), and PDSS2 (*r* = 0.60, *p* < 0.001)	

The Parkinson's Disease Questionnaire (PDQ)	Tan et al. [54]	Patients who were diagnosed PD by neurologist (*n* = 88)	Internal consistency:	Spearman's rho shows all items to own-dimension correlations >0.4 except for one item in “bodily discomfort” Factor loadings of PDQ-39 = 0.50–0.79, indicating strong correlation between items and loadings	Vague
			Full Scale (except Bodily Discomfort): *α* = 0.74–0.94		
			PDQ-39 test-retest reliability = 0.67–0.87		
			PDQ-39SI test-retest reliability = 0.85		

Modified Fatigue Impact Scale (MFIS)	Schiehser et al. [55]	Patients who were (1) determined nondemented by Diagnostic and Statistical Manual of Mental Disorders-IV criteria; (2) MDRS cutoff score of ≥124 (*n* = 100)	Internal consistency:	Covergent validity: Strong correlation between the MFIS total score and PANAS-X (rs = 0.585, *p* < 0.001), STAI-state (rs = 0.518, *p* < 0.001), HAM-D (rs = 0.497, *p* < 0.001), GDS (rs = 0.599, *p* < 0.001), and AS (rs = 0.564, *p* < 0.001)	Yes
			Full scale: *α* = 0.96		
			Cognitive subscale: *α* = 0.95		
			Physical/social subscale: *α* = 0.95		

Multidimensional Fatigue Inventory (MdFI)	Elbers et al. [17] Lou et al. [56]	Patients who are (1) age 18–80; (2) diagnosis of PD via UK Brain Bank Criteria; (3) Hoehn and Yahr stage II–IV (*n* = 153) Patients who are (1) Hoehn and Yahr stage < IV; (2) had at least two of the four cardinal symptoms for PD (tremor, rigidity, bradykinesia, and postural instability) (*n* = 39)	Internal consistency: subscales:	Structural validity: PCA results show that all 20 MdFI items had unique loading of ≥0.40. Moderate correlations between factor 1 (GF/PF) and factor 2 (MF) (*r* = 0.35), between factor 1 (GF/PF) and factor 4 (RA) (*r* = 0.44), and between factor 3 (RM) and factor 4 (RA) (*r* = 0.38) Convergent validity: Established validity with FSI, POMS fatigue scale, VAS-energy, VAS-fatigue (VAS-f), D-FIS, and Global Perception of Fatigue (all *p* < 0.01)	Yes
			MdFI total: *α* = 0.92, ICC (95% CI) = 0.80 (0.70–0.87)		
			GF: *α* = 0.79, ICC (95% CI) = 0.73 (0.61–0.82)		
			PF: *α* = 0.83, ICC (95% CI) = 0.81 (0.71–0.87)		
			Ra: *α* = 0.88, ICC (95% CI) = 0.74 (0.62–0.83)		
			MF: *α* = 0.86, ICC (95% CI) = 0.65 (0.50–0.76) reduced motivation: *α* = 0.74, ICC (95% CI) = 0.79 (0.69–0.86)		
			N/A		

Visual Analog Scale for Fatigue (VAS-F)	Lee et al. [57] Tseng et al. [58]	Healthy individuals (*n* = 75) and a sample of patients medically evaluated for sleep disorders (*n* = 57) Patients who (1) have a diagnosis of stroke ≥6 months and ≤5 years ago; (2) score <2 on a dementia screening tool AD8 (*n* = 21)	Internal consistency:	Pearson's *r* shows strong association between VAS-F fatigue and energy subscale N/A	No
			Fatigue subscale: *α* = 0.91–0.96		
			Energy subscale: *α* = 0.94–0.96		
			Correlations of subscales: −0.54 for the normal group and −0.73 for the patient group		
			ICC:		
			At rest: 0.851 (CI = 95%, 0.673–0.936, *p* < 0.001)		
			Immediately after exercise: 0.851 (CI = 95%, 0.663–0.934, *p* < 0.001)		
			15 minutes after exercise: 0.851 (CI = 95%, 0.749–0.953, *p* < 0.001)		

Multicomponent Fatigue Index (McFI)	Rabo et al. [15]	Filipino patients who are (1) diagnosed to have idiopathic PD via Parkinson's Data Bank Criteria; (2) modified Hoehn and Yahr stage of ≤3	N/A	Unpaired *t*-test shows all the scores of PD patients were statistically significant when compared with the normal control and all of the five dimensions in the McFI showed a higher score	No

Brief Fatigue Inventory (BFI)	Radbruch et al. [59] Okuyama et al. [60]	Patients with chronic cancer-related pain (*n* = 22) and noncancer-related pain (*n* = 95) Japanese patients who (1) are pathologically diagnosed of cancer; (2) not suffering from severe cognitive disorders (*n* = 252)	Test-retest: 0.79–0.91 Internal consistency: *α* = 0.89–0.96	Criterion validity: Spearman's rho shows strong correlation with SF-36, and ECOG performance status, and MIDOS	No
				Convergent validity: strong correlations with POMS fatigue (*r* = 0.60–0.70)	
				Construct validity: Factor analysis verified one factor	
				Discriminant validity: distinguished fatigue severity between patients with poor and good performance status	

Fatigue Severity Inventory (FSI)	Rabo et al. [15] Lou et al. [56]	Patients with chronic cancer-related pain (*n* = 22) and noncancer-related pain (*n* = 95) Patients who are (1) Hoehn and Yahr stage < IV; (2) had at least two of the four cardinal symptoms for PD (tremor, rigidity, bradykinesia, and postural instability) (*n* = 39)	N/A N/A	^*∗*^See validity for Multicomponent Fatigue Index (McFI)	No
				See convergent validity for Multidimensional Fatigue Inventory (MdFI)	
				Convergent validity: strong correlation with the McFI (*p* < 0.001), but didn't provide coefficient value	

Neuro-QoL item bank v1.0 (Neuro-QoL)	Nowinski et al. [61]	PD patients who are (1) age of 18+; (2) Hoehn and Yahr stage < V (*n* = 120)	Internal consistency:	Convergent validity: Pearson's *r* shows strong correlation between Neuro-QoL and PD validation measures. Measures of emotional health are statistically significantly correlated with measures of similar constructs. Moderate correlation with PDQ-39 Discriminant validity: patients in H&Y stage I and II scored significantly better than those in stages III and IV for all items except applied cognition–General concerns and emotional and behavioral dyscontrol	Yes
			Full scale: *α* = 0.81–0.94		
			Test-retest reliability: ICC = 0.66 to 0.80		

Functional Assessment of Chronic Illness Therapy-Fatigue Scale (FACIT-F)	Hagell et al. [62]	Swedish patients who are (1) age 54–74; (2) Hoehn and Yahr stage II-III (*n* = 118)	Internal consistency:	Floor (1.7%) and ceiling effects (0%) are minimal	Vague
			Full scale: *α* = 0.90–0.92	Construct validity: strong correlation (*r* = −0.77) with NHP-EN, PFS (*r* = −0.89), and FSS (*r* = 0.71)	
			Test–retest reliability = 0.85	Discriminant validity: significant ability to discriminated between fatigue and nonfatigue patients	

Fatigue Impact Scale (FIS)	Martinez-Martin et al. [63]	Patients who are with (1) diagnosis of PD as per the United Kingdom PD Society Brain Bank criteria; (2) age of 39+; (3) Hoehn and Yahr classification stages I–IV	Internal consistency:	Factor analysis shows 69.5% of the variance explained by a single factor	Yes
			Full scale: *α* = 0.93	Convergent validity: statistical significant mild-moderate correlation with McFI-GF (*r* = 0.55), VAS-F (*r* = 0.62), and GPF (*r* = 0.54). Good correlation between global fatigue and physical fatigue, depression, and disability measures	
			Interitem correlation: 0.48 (items 1–4)-0.78 (items 3–8)	Discriminant validity: scores are significantly different between patients with no/mild fatigue (GPF = 0–2) and those with moderate/severe fatigue (GPF = 3–5)	

Nottingham Health Profile (NHP)	Hagell et al. [64]	Swedish PD patients (*n* = 81)	Internal consistency: Full scale: *α* = 0.63 (SL)-0.9 (SI)	Substantial floor effects (≥20%) observed in all NHP-EN scales, which decreased with increasing PD severity	No
				Spearman's rho shows moderate correlations between NHP score and patient QoL (rs = −0.497–−0.650), which are similar to those between perceived overall QoL and indices of PD severity	

Fatigue Assessment Inventory (FAI)	Friedman and Friedman [6]	Patients with presumed idiopathic PD (*n* = 58)	Internal consistency:	Convergent validity: Moderate correlations (*r* = −0.68) with VAS-F	No
			Full scale (question except 15, 16, 19): *α* = 0.61–1		
			Full scale (question 15, 16, 19): *α* = 0.27		

^1^The reliability, validity, and the present of mental fatigue subscale are recorded for scale evaluation.

**Table 4:** Application of fatigue scales^1^.

Fatigue scale	Language modification	Neurobiology mechanism	Combinations of scale usage
Fatigue Severity Scale (FSS)	Portuguese, Turkish	Motor symptoms, cortical functions, autonomic dysfunction [66]	FSS, PFS: 2
			FSS, PDQ: 4
			FIS, FSS: 1
			MFIS, PDQ, FSS, MFIS: 1
			FAI, VAFS, FSS: 1
			MFIS, FSS, PFS: 2
			PFS, FSS, PDQ: 1
			FACIT-F, FSS, PFS, VAFS, MFIS, PDQ: 1
			MFIS, FSS: 1
			FSS, NHP: 1
Parkinson's Fatigue Scale (PFS)	Japanese, Spanish, Turkish, Chinese	Neuropsychiatric triad, bilateral subthalamic nucleus (STN)	PFS, FSS: 2
			PFS, PDQ: 3
			PFS, FSS, MFIS: 2
			PFS, MFIS: 1
			PFS, FSS, PDQ: 1
The Parkinson's Disease Questionnaire (PDQ)	—	—	PDQ, FSS: 4
			PDQ, PFS: 3
			PDQ, PFS, FSS: 1
			MdFI, FSS, MFIS, PDQ: 1
			FACIT-F, FSS, PFS, VAFS, MFIS, PDQ: 1
			PDQ, MFIS: 1
Modified Fatigue Impact Scale (MFIS)	Brazilian, Portuguese	Conventional subthalamic nucleus (STN), combined subthalamic nucleus and substantia nigra (STN + SNr)	MdFI, FSS, MFIS: 1
			MFIS, FSS, PFS: 2
			MFIS, PFS: 1
			FACIT-F, FSS, PFS, VAFS, MFIS, PDQ: 1
			MFIS, PDQ: 1
			MFIS, FSS: 1
Multidimensional Fatigue Inventory (MdFI)	Dutch	—	MdFI, FSS, MFIS, PDQ: 1
			MdFI, FSS: 1
Visual Analog Scale for Fatigue (VAS-F)	—	—	FAI, VAFS, FSS: 1
			FACIT-F, FSS, PFS, VAFS, MFIS, PDQ: 1
Multicomponent Fatigue Index (McFI)	—	—	McFI, FSS: 1
Brief Fatigue Inventory (BFI)	—	Hallucination	—
Fatigue Symptom Inventory (FSI)	—	—	FSI, McFI: 1
Neuro-QoL Item Bank v1.0 (Neuro-QoL)	—	—	—
Functional Assessment of Chronic Illness Therapy-Fatigue Scale (FACIT-F)	—	—	FACIT-F, FSS, PFS, VAFS, MFIS, PDQ: 1
Fatigue Impact Scale (FIS)	Turkish	—	FIS, FSS: 1
Nottingham Health Profile (NHP)	—	—	NHP, FSS: 1
Fatigue Assessment Instrument (FAI)	—	—	FAI, VAFS, FSS: 1

^1^Among all articles, the applications of scales in research/clinical practice are recorded including language modification/translation, neurobiology mechanism, and combinations of scale usage. FSS, PFS, MFIS, MdFI, and FIS are found to have been proven to be valid and reliable after translation. FSS, PFS, MFIS, and BFI are related to certain pathophysiological basis of mental fatigue in PD. Almost every scale is coupled with other scales.

## Data Availability

All data are retrieved from PubMed, Scopus, and Embase, reviewed via PRISMA guidelines. The literature data used to support the findings of this study are included within the article.

## Appendix

### A. Fatigue Severity Scale (FSS)

As the most frequently used fatigue measure, Fatigue Severity Scale (FSS) has been recorded in more than half of the sample articles. FSS originally aimed to test the functional impact of fatigue in Multiple Sclerosis. It is a self-administered fatigue rating scale that has nine items, each scoring on a seven-grade Likert scale of 1 (strongly disagree) to 7 (strongly agree) and with 3 types of cutoff points (*x̄* > 5, *x̄* ≥ 4, *x̄* > 4). By evaluating patients' self-reported scores, FSS measures physical and mental fatigue intensity in I–III Hoehn and Yahr Stages. The total FSS score is the mean score of all nine items, with a higher score conveying higher severity of fatigue. Although FSS does not define fatigue and differentiate its subdomains specifically, its psychometric properties have been tested and validated across various chronic diseases like PD and MS. Due to its simplicity and strong psychometric properties, it has been translated into various languages and widely used by researchers to assess numerous diseases [70].

### B. Parkinson's Fatigue Scale (PFS)

Parkinson's Fatigue Scale (PFS) has been recorded in 17 sample articles. PFS aims to test solely the impact of physical fatigue on aspects of daily functioning of PD patients. It is a self-administered fatigue scale that has 16 items, each scoring from 1 (strongly disagree) to 5 (strongly agree) and with 2 types of cutoff points (qualitative: *x̄* ≥ 8, quantitative: *x̄* ≥ 5). Among all the items, nine items aim to test the effects of fatigue on daily activities, while seven target the degree of physical fatigue via patients' subjective observations. PFS measures physical and mental fatigue intensity in I–V Hoehn and Yahr Stages by evaluating patients' self-reported scores. There are three scoring methods for PFS: (1) calculate the total PFS score by average the score of all 16 items, (2) calculate binarily by ranging the total PFS from 0 to 16, with one point added for every “agree” or “strongly agree” response, and (3) calculate the total PFS by summing the score of all 16 items. Due to its specificity towards PD and good psychometric properties, PFS has been widely used to measure fatigue severity among PD patients [71].

### C. Parkinson's Disease Questionnaire (PDQ)

Parkinson's Disease Questionnaire (PDQ) has been recorded in 11 sample articles. PDQ assesses PD's impact on eight dimensions of daily functioning, with fatigue being one of the major dimensions. It is a self-administered fatigue rating scale with short and long forms including 8 and 39 items respectively, each scoring from 1 (never) to 5 (always) and uses percentile scores as cutoff points. Compared with unidimensional FSS and PFS, PDQ evaluates patients' self-reported scores and measures patients' mobility and mental fatigue intensity, quality of life, and depression in I–V Hoehn and Yahr Stages, with lower scores reflecting better quality of life. The dimension score is calculated by converting the sum of score into percentile, and the total score, often summarized by PDF-39 SI, is calculated by summing the dimensional scores and dividing it by eight [72].

### D. Modified Fatigue Impact Scale (MFIS)

Modified Fatigue Impact Scale (MFIS) has been recorded in 8 sample articles. Its main purpose is to measure physical, mental, and psychosocial functioning by evaluating patients' self-reported scores. MFIS is a multidimensional, self-administered fatigue rating scale with a short and long form that include 5 and 21 items, respectively, each scoring from 0 (never) to 4 (always) and with the cutoff points of 38 in total. Each question is scored on a 5-grade Likert scale. The total score of MFIS is calculated by summing the scores of all 21 items. Individual subscale scores can be calculated congruently. MFIS is originally designed to assess MS patients and has developed multiple language modifications [73].

### E. Multidimensional Fatigue Inventory (MdFI)

Multidimensional Fatigue Inventory (MdFI) has been recorded in 5 sample articles. It includes 20 items, each scoring from 1 (yes, that is true) to 5 (no, that is not true). As a multidimensional, self-reported scale, MdFI measures the fatigue severity and impacts from five dimensions: general fatigue (GF), physical fatigue (PF), mental fatigue (MF), reduced motivation (RM), and reduced activity. Each dimension contains four items, with two items convey positive direction and two convey negative direction. Total score for full scale and individual dimensions are calculated by summing up all the items indicative of fatigue. Although originally written in Norwegian, there are multiple language modification for MdFI cross various diseases, including PD [74].

### F. Visual Analog Scale for Fatigue (VAS-F)

Visual Analog Scale for Fatigue (VAS-F) has been recorded in 2 sample articles. It is often used to cross-validate results of other PD fatigue scales. Unlike other scales included in this review, VAS-F is designed to measure subjective phenomenon put asking the subjects to mark a straight line to approximate their degree of sensation about a set variable. It includes 18 items, each scoring from 0 (not at all) to 10 extremely and uses percentile scores as cutoff points. As a bidimensional scale, VAS-F measures physical and mental fatigue intensity and energy by evaluating patients' self-reported scores. There is a limited record for the reliability of VAS-F, which may count as the weakness of the scale [57].

### G. Multicomponent Fatigue Index (McFI)

Multicomponent Fatigue Index (McFI) is an uncommon scale that has only been recorded in 1 sample article. Its forms for physical fatigue and mental fatigue include 7 and 8 items respectively, each scoring from 1 (not at all) to 5 (a great deal). The cutoff points cannot be accessed. As a unidimensional scale, McFI measures physical and mental fatigue severity by evaluating patients' self-reported scores. The reliability of McFI cannot be found in recent studies, which may be the reason that McFI is derived from current fatigue scales [75].

### H. Brief Fatigue Inventory (BFI)

Brief Fatigue Inventory (BFI) has been recorded in 1 sample article. Originally designed to measure the severity and impact of fatigue quickly and efficiently in cancer patients, BFI includes 9 items, each scoring from 0 (no fatigue) to 10 (as bad as you can imagine), with cutoff points being 36 in total. As a unidimensional scale, BFI measures physical and mental fatigue intensity by evaluating patients' self-reported scores. The total score for full scale is calculated by averaging all the items in the scale (The University of Texas M.D. Anderson Cancer Center, 1997). Originally written in English, BFI now has language modified versions in Japanese, Korean, Philippine, Greek, German, and Taiwan versions [65].

### I. Fatigue Severity Inventory (FSI)

Fatigue Symptom Inventory (FSI) has been recorded in 1 sample article. Having derived from FSS and FAI, FSI is a questionnaire that includes 33 items, each scoring from 0 (not at all fatigued) to 10 (as fatigued as I could be), with cutoff points being 42 in total. Despite being originally designed for MS, FSI has been adapted for PD. As a unidimensional scale, FIS measures physical and mental fatigue severity by evaluating patients' self-reported scores [76]. No language modification is found.

### J. Neuro-QoL Item Bank v1.0 (Neuro-QoL)

Neuro-QoL item Bank v1.0 (Neuro-QoL) has been recorded in 1 sample article. Created to survey aspects of subjects' quality of life, Neuro-QoL has 4 types of forms, including adult bank, adult short form, pediatric, and pediatric short form with corresponding item numbers of 10–45, 5–9, 1–20, 8–10. The scores of each item ranges from 1 (cannot do) to 5 (none) on a 5-grade Likert scale. Total score is calculated via Neuro-QoL user manual for each short form, The cutoff points cannot be accessed. As a multidimensional scale, Neuro-QoL measures fatigue intensity, activities of daily living, attention and working memory, behavior, mental, communication, depression, functional mobility, mental health, occupational performance, life participation, pain, negative and positive effects, quality of life, sleep, relationships, stress, and social support by evaluating patients' self-reported scores and by interview. The questionnaire is adapted and validated to various diseases, including PD, based on different research focus and ethics [77, 78].

### K. Functional Assessment of Chronic Illness Therapy-Fatigue Scale (FACIT-F)

Functional Assessment of Chronic Illness Therapy-Fatigue Scale (FACIT-F) has been recorded in 1 sample article. It was developed to measure anemia-related fatigue in cancer patients. FACIT-F includes 13 items, each scoring from 0 (not at all) to 4 (very much). The cutoff points cannot be accessed. Ranging from 0 to 52, the FACIT-F total score is calculated with its own algorithm. As a unidimensional scale, FACIT-F measures physical and mental fatigue intensity by evaluating patients' self-reported scores. However, no clear definition of fatigue was provided by the scale. Being one of the most popular fatigue-assessing instruments, FACIT-F has been validated to many diseases, including PD, and has numerous language modifications [79].

### L. Fatigue Impact Scale (FIS)

Fatigue Impact Scale (FIS) has been recorded in 1 sample article. Designed to assess the severity of PD-induced fatigue and its impact on daily functioning, FIS includes 40 items, each scoring from 0 (no problem) to 4 (extreme problem) on a 5-grade Likert scale. The cutoff points cannot be accessed. As a unidimensional scale, FIS measures physical and mental fatigue intensity by evaluating patients' self-reported scores [80]. There is no report of validated language modification for FIS in the context of PD.

### M. Nottingham Health Profile (NHP)

Nottingham Health Profile (NHP) has been recorded in 1 sample article. Intended as a multidimensional assessment that measures the medical and social interventions of physical mobility, pain, sleep, social isolation, emotional reactions, and energy level with health problems and disease, NHP includes 38 items, each scoring as yes/no on a dichotomous scale, and uses weighted percentages as cutoff points. Items within each of the six categories are rated according to relative importance. When calculating scores, items are rescaled so they can vary between 0 and 100 within individual sections. The second part of NHP explores how the health problem has impacted seven areas of his life: employment, household activities, social life, home life, sex life, hobbies and interests, and holidays. With wide application in Europe, NHP has versions in many languages and has been adapted to various diseases, including PD [81].

### N. Fatigue Assessment Inventory (FAI)

Fatigue Assessment Instrument (FAI) has been recorded in 1 sample article. Derived from FSS, FAI includes 29 items, each scoring from 1 (completely disagree) to 7 (completely agree) on a 7-grade Likert scale. However, unlike FSS, FAI provides a stated definition of fatigue. For all the items, the cutoff points cannot be accessed. The total and subscale scores for FAI are calculated by taking the mean of all items score, with higher score indicating more severe fatigue. As a multidimensional scale, FAI measures severity, situation-specificity, psychological consequences, sleep, and mental and physical fatigue intensity [82]. There is generally a lack of systematic evaluation on the reliability and validity of FAI in the context of PD.

## References

[1] Hindeya Gebreyesus H., Gebrehiwot Gebremichael T. (2020). The potential role of astrocytes in Parkinson’s disease (PD). *Medical Science*.

[2] Zhong Q. Q., Zhu F. (2022). Trends in prevalence cases and disability-adjusted life-years of Parkinson’s disease: findings from the global burden of disease study 2019. *Neuroepidemiology*.

[3] Ruiz P. J. G., Catalán M. J., Carril J. F. (2011). Initial motor symptoms of Parkinson disease. *The Neurologist*.

[4] Moustafa A. A., Chakravarthy S., Phillips J. R. (2016). Motor symptoms in Parkinson’s disease: a unified framework. *Neuroscience and Biobehavioral Reviews*.

[5] Pfeiffer R. F. (2016). Non-motor symptoms in Parkinson’s disease. *Parkinsonism and Related Disorders*.

[6] Friedman J., Friedman H. (1993). Fatigue in Parkinson’s disease. *Neurology*.

[7] Havlikova E., Rosenberger J., Nagyova I. (2008). Clinical and psychosocial factors associated with fatigue in patients with Parkinson’s disease. *Parkinsonism and Related Disorders*.

[8] Friedman J. H., Abrantes A., Sweet L. H. (2011). Fatigue in Parkinson’s disease. *Expert Opinion on Pharmacotherapy*.

[9] Park A., Stacy M. (2009). Non-motor symptoms in Parkinson’s disease. *Journal of Neurology*.

[10] Siciliano M., Trojano L., Santangelo G., De Micco R., Tedeschi G., Tessitore A. (2018). Fatigue in Parkinson’s disease: a systematic review and meta-analysis. *Movement Disorders*.

[11] Chaudhuri K. R., Healy D. G., Schapira A. H. (2006). Non-motor symptoms of Parkinson’s disease: diagnosis and management. *The Lancet Neurology*.

[12] Moher D., Liberati A., Tetzlaff J., Altman D. G. (2009). Preferred reporting items for systematic reviews and meta-analyses: the PRISMA statement. *Journal of Clinical Epidemiology*.

[13] Alves G., Wentzel-Larsen T., Larsen J. P. (2004). Is fatigue an independent and persistent symptom in patients with Parkinson disease?. *Neurology*.

[14] Goulart F. O., Godke B. A., Borges V. (2009). Fatigue in a cohort of geriatric patients with and without Parkinson’s disease. *Brazilian Journal of Medical and Biological Research*.

[15] Rabo C. S., Rosales R. L., Corrales M. L. (2009). The occurrence of fatigue in independent and clinically stable Filipino patients with idiopathic Parkinson’s disease. *Journal of Movement Disorders*.

[16] Chou K. L., Persad C. C., Patil P. G. (2012). Change in fatigue after bilateral subthalamic nucleus deep brain stimulation for Parkinson’s disease. *Parkinsonism and Related Disorders*.

[17] Elbers R. G., van Wegen E. E., Verhoef J., Kwakkel G. (2012). Reliability and structural validity of the Multidimensional Fatigue Inventory (MFI) in patients with idiopathic Parkinson’s disease. *Parkinsonism and Related Disorders*.

[18] Valderramas S., Feres A. C., Melo A. (2012). Reliability and validity study of a Brazilian-Portuguese version of the fatigue severity scale in Parkinson’s disease patients. *Arq Neuropsiquiatr*.

[19] Sáez-Francàs N., Hernández-Vara J., Corominas Roso M., Alegre Martín J., Casas Brugué M. (2013). The association of apathy with central fatigue perception in patients with Parkinson’s disease. *Behavioral Neuroscience*.

[20] Stocchi F. (2014). Benefits of treatment with rasagiline for fatigue symptoms in patients with early Parkinson’s disease. *European Journal of Neurology*.

[21] Tanaka K., Wada-Isoe K., Yamamoto M. (2014). Clinical evaluation of fatigue in Japanese patients with Parkinson’s disease. *Brain and Behavior*.

[22] Cochrane G. D., Rizvi S., Abrantes A. M., Crabtree B., Cahill J., Friedman J. H. (2015). The association between fatigue and apathy in patients with either Parkinson’s disease or multiple sclerosis. *Parkinsonism and Related Disorders*.

[23] Skorvanek M., Gdovinova Z., Rosenberger J. (2015). The associations between fatigue, apathy, and depression in Parkinson’s disease. *Acta Neurologica Scandinavica*.

[24] Ikeda M., Kataoka H., Ueno S. (2016). Fatigue is associated with the onset of hallucinations in patients with Parkinson’s disease: a 3-year prospective study. *eNeurologicalSci*.

[25] Paul B. S., Singh A., Jain D. (2016). Assessment of fatigue in Parkinson’s disease: Indian perspective. *Annals of Indian Academy of Neurology*.

[26] Tessitore A., Giordano A., De Micco R. (2016). Functional connectivity underpinnings of fatigue in Drug-Naïve patients with Parkinson’s disease. *Movement Disorders*.

[27] Hidding U., Gulberti A., Horn A. (2017). Impact of combined subthalamic nucleus and substantia nigra stimulation on neuropsychiatric symptoms in Parkinson’s disease patients. *Parkinson’s Disease*.

[28] Chong R., Albor L., Wakade C., Morgan J. (2018). The dimensionality of fatigue in Parkinson’s disease. *Journal of Translational Medicine*.

[29] Olivola E., Brusa L., Rocchi C. (2018). Does fatigue in Parkinson’s disease correlate with autonomic nervous system dysfunction?. *Neurological Sciences*.

[30] Öztürk E. A., Gönenli Koçer B., Umay E., Çakcı A. (2018). Turkish version of Parkinson Fatigue Scale: validity and reliability study of binary scoring method. *Turkish Journal of Physical Medicine and Rehabilitation*.

[31] Yang Z.-J. L., Wang Y.-Q., Zhang Z.-S., Fang H., Cui G.-Y. (2018). Characteristics of fatigue and correlation with other symptoms of Parkinson’s disease. *Chinese Journal of Contemporary Neurology and Neurosurgery*.

[32] Atan T., Özyemi̇şci̇ Taşkiran Ö, Bora Tokçaer A., Kaymak Karataş G., Karakuş Çalışkan A., Karaoğlan B. (2019). Effects of different percentages of body weight-supported treadmill training in Parkinson’s disease: a double-blind randomized controlled trial. *Turkish Journal of Medical Sciences*.

[33] Çilga G., Genç A., Çolakoğlu B. D., Kahraman T. (2019). Turkish adaptation of Parkinson fatigue scale and investigating its psychometric properties. *International Journal of Rehabilitation Research*.

[34] Martinez-Martin P., Wetmore J. B., Arbelo J. M., Catalán M. J., Valldeoriola F., Rodriguez-Blazquez C. (2019). Validation study of the Parkinson Fatigue Scale in advanced Parkinson disease. *Patient Related Outcome Measures*.

[35] Nguyen T. D., Tran Q. V., Kim Huynh K. (2019). An assessment of the effectiveness of three-month management of idiopathic Parkinson’s disease (I-PD) with levodopa (L-DOPA) to treat fatigue severity, HRQOL and cortical dysfunctions. *Journal of Critical Reviews*.

[36] Niimi Y., Shima S., Mizutani Y., Ueda A., Ito S., Mutoh T. (2019). Fatigue evaluated using the 16-item Parkinson Fatigue Scale (PFS-16) predicts Parkinson’s disease prognosis. *Fujita Medical Journal*.

[37] Abasi A., Raji P., Friedman J. H. (2020). Effects of vestibular rehabilitation on fatigue and activities of daily living in people with Parkinson’s disease: a pilot randomized controlled trial study. *Parkinson’s Disease*.

[38] Ahn J. H., Song J., Lee D. Y., Youn J., Cho J. W. (2021). Understanding fatigue in progressive supranuclear palsy. *Scientific Reports*.

[39] Lopes J., AraÚjo H., Smaili S. M. (2020). Fatigue in Parkinson’s disease: Brazilian validation of the modified fatigue impact scale. *Arq Neuropsiquiatr*.

[40] Singh G., Jain T., Liu W. (2020). Effects of balance training on nonmotor symptoms in individuals with Parkinson disease. *Topics in Geriatric Rehabilitation*.

[41] Yu H. X., Guo M. R., Li G., Zhang B. (2020). Association between fatigue and motor progression in Parkinson’s disease in southern Chinese. *Neurological Sciences*.

[42] Cao X. Y., Zhang J. R., Shen Y. (2021). Fatigue correlates with sleep disturbances in Parkinson disease. *Chinese Medical Journal*.

[43] Hill H. M., Swink L. A., Atler K. E., Anderson A. K., Fling B. W., Schmid A. A. (2020). Merging Yoga and Occupational Therapy for Parkinson’s Disease improves fatigue management and activity and participation measures. *British Journal of Occupational Therapy*.

[44] Lee D. G., Lindsay A., Yu A. (2021). Data-driven prediction of fatigue in Parkinson’s disease patients. *Front Artif Intell*.

[45] Lin I., Edison B., Mantri S. (2021). Triggers and alleviating factors for fatigue in Parkinson’s disease. *PLoS One*.

[46] Alizadeh N., Packer T. L., Sturkenboom I., Eskes G., Warner G. (2022). Managing fatigue in Parkinson’s disease: protocol for a pilot randomized controlled trial. *Canadian Journal of Occupational Therapy*.

[47] Paul B., Bansal N., Paul G., Singh G. (2022). Gender differences and impact of autonomic disturbance on fatigue and quality of life in Parkinson’s disease. *Neurology India*.

[48] Béreau M., Castrioto A., Lhommée E. (2022). Fatigue in de novo Parkinson’s Disease: Expanding the Neuropsychiatric Triad?. *Journal of Parkinson’s Disease*.

[49] Lawrie S., Coe S., Mansoubi M. (2023). Dietary patterns and nonmotor symptoms in Parkinson’s disease: a cross-sectional analysis. *Journal of the American Nutraceutical Association*.

[50] Siciliano M., Kluger B., De Micco R. (2022). Validation of new diagnostic criteria for fatigue in patients with Parkinson disease. *European Journal of Neurology*.

[51] Fereshtehnejad S. M., Hadizadeh H., Farhadi F., Shahidi G. A., Delbari A., Lökk J. (2013). Reliability and validity of the Persian version of the fatigue severity scale in idiopathic Parkinson’s disease patients. *Parkinson’s Disease*.

[52] Hagell P., Nilsson M. H. (2009). The 39-item Parkinson’s disease questionnaire (PDQ-39): is it a unidimensional construct?. *Ther Adv Neurol Disord*.

[53] Dagklis I. E., Tsantaki E., Kazis D. (2019). The Parkinson fatigue scale: an evaluation of its validity and reliability in Greek Parkinson’s disease patients. *Neurological Sciences*.

[54] Tan L. C., Luo N., Nazri M., Li S. C., Thumboo J. (2004). Validity and reliability of the PDQ-39 and the PDQ-8 in English-speaking Parkinson’s disease patients in Singapore. *Parkinsonism and Related Disorders*.

[55] Schiehser D. M., Ayers C. R., Liu L., Lessig S., Song D. S., Filoteo J. V. (2013). Validation of the modified fatigue impact scale in Parkinson’s disease. *Parkinsonism and Related Disorders*.

[56] Lou J. S., Kearns G., Oken B., Sexton G., Nutt J. (2001). Exacerbated physical fatigue and mental fatigue in Parkinson’s disease. *Movement Disorders*.

[57] Lee K. A., Hicks G., Nino-Murcia G. (1991). Validity and reliability of a scale to assess fatigue. *Psychiatry Research*.

[58] Tseng B. Y., Gajewski B. J., Kluding P. M. (2010). Reliability, responsiveness, and validity of the visual analog fatigue scale to measure exertion fatigue in people with chronic stroke: a preliminary study. *Stroke Research and Treatment*.

[59] Radbruch L., Sabatowski R., Elsner F., Everts J., Mendoza T., Cleeland C. (2003). Validation of the German version of the brief fatigue inventory. *Journal of Pain and Symptom Management*.

[60] Okuyama T., Wang X. S., Akechi T. (2003). Validation study of the Japanese version of the brief fatigue inventory. *Journal of Pain and Symptom Management*.

[61] Nowinski C. J., Siderowf A., Simuni T., Wortman C., Moy C., Cella D. (2016). Neuro-QoL health-related quality of life measurement system: validation in Parkinson’s disease. *Movement Disorders*.

[62] Hagell P., Höglund A., Reimer J. (2006). Measuring fatigue in Parkinson’s disease: a psychometric study of two brief generic fatigue questionnaires. *Journal of Pain and Symptom Management*.

[63] Martinez-Martin P., Catalan M. J., Benito-Leon J. (2006). Impact of fatigue in Parkinson’s disease: the fatigue impact scale for daily use (D-FIS). *Quality of Life Research*.

[64] Hagell P., Whalley D., McKenna S. P., Lindvall O. (2003). Health status measurement in Parkinson’s disease: validity of the PDQ-39 and Nottingham Health Profile. *Movement Disorders*.

[65] Bland J. M., Altman D. G. (1997). Statistics notes: Cronbach’s alpha. *BMJ*.

[66] Ahn J. H., Kim M., Mun J. K. The dysfunctional autonomic function and dysfunctional fatigue in drug naïve Parkinson’s disease. *Journal of Parkinson’s Disease*.

[67] Lauren K. (2003). Fatigue (the most common complaints series). https://www.amazon.in/Fatigue-Common-Complaints-Lauren-Krupp/dp/075067038X.

[68] Chaudhuri A., Behan P. O. (2000). Fatigue and basal ganglia. *Journal of the Neurological Sciences*.

[69] Muangpaisan W., Mathews A., Hori H., Seidel D. (2011). A systematic review of the worldwide prevalence and incidence of Parkinson’s disease. *Medical Journal of the Medical Association of Thailand*.

[70] Fss Fatigue severity scale (FSS). https://www.mercy.net/content/dam/mercy/en/pdf/fatigue-severity-scale-epworth-sleepiness-scale-questionaire.pdf.

[71] Brown R. G. (2004). *Parkinson’s Disease Fatigue Scale*.

[72] Sra (2017). *Parkinson’s Disease Questionnaire*.

[73] Sra (2017). Modified fatigue impact scale (MFIS). https://www.sralab.org/sites/default/files/2017-06/mfis.pdf.

[74] Smets E. M. A., Garssen B., Bonke B., De Haes J. C. J. M. (1995). The multidimensional Fatigue Inventory (MFI) psychometric qualities of an instrument to assess fatigue. *Journal of Psychosomatic Research*.

[75] Paul R. H., Beatty W. W., Schneider R., Blanco C. R., Hames K. A. (1998). Cognitive and physical fatigue in multiple sclerosis: relations between self-report and objective performance. *Applied Neuropsychology*.

[76] Hann D. M., Denniston M. M., Baker F. (2000). Measurement of fatigue in cancer patients: further validation of the fatigue symptom inventory. *Quality of Life Research: An International Journal of Quality of Life Aspects of Treatment, Care and Rehabilitation*.

[77] PhoobotNIfNDa S. (2014). Neuro-qol scale v1.0- communication- short form. https://www.iconquerms.org/sites/all/files/attachments/pdfs/FullNeuroQoL.pdf.

[78] Cella D., Lai J. S., Nowinski C. J. (2012). Neuro-QOL: brief measures of health-related quality of life for clinical research in neurology. *Neurology*.

[79] Ser (2015). *FACIT Fatigue Scale*.

[80] Shahid A., Wilkinson K., Marcu S., Shapiro C. M., Shahid A., Wilkinson K., Marcu S., Shapiro C. M. (2012). Fatigue assessment inventory (FAI). *STOP, THAT and One Hundred Other Sleep Scales*.

[81] Conceptuel R. (2001). Nottingham health profile. https://www.physio-pedia.com/Nottingham_Health_Profile.

[82] Shahid A., Wilkinson K., Marcu S., Shapiro C. M., Shahid A., Wilkinson K., Marcu S., Shapiro C. M. (2012). Fatigue impact scale (FIS). *STOP, THAT and One Hundred Other Sleep Scales*.

